# Functional Analysis of Human Pathological Semen Samples in an Oocyte Cytoplasmic Ex Vivo System

**DOI:** 10.1038/s41598-018-33468-x

**Published:** 2018-10-18

**Authors:** Farners Amargant, Désirée García, Montserrat Barragán, Rita Vassena, Isabelle Vernos

**Affiliations:** 1 Cell and Developmental Biology Programme, Centre for Genomic Regulation (CRG), The Barcelona Institute of Science and Technology, Dr. Aiguader 88, Barcelona, 08003, Spain; 2 Clínica EUGIN, Travessera de les Corts 322, Barcelona, 08029 Spain; 3 Fundació EUGIN, Travessera de les Corts 314, Barcelona, 08029 Spain; 4 Institució Catalana de Recerca I Estudis Avançats (ICREA), Pg. Lluis Companys 23, Barcelona, 08010, Spain; 5 Universitat Pompeu Fabra (UPF), Barcelona, Spain

## Abstract

Human fertilization and embryo development involve a wide range of critical processes that determine the successful development of a new organism. Although Assisted Reproduction Technologies (ART) may help solve infertility problems associated to severe male factor, the live birth rate is still low. A high proportion of ART failures occurs before implantation. Understanding the causes for these failures has been difficult due to technical and ethical limitations. Diagnostic procedures on human spermatozoa in particular have been limited to morphology and swimming behaviours while other functional requirements during early development have not been addressed due to the lack of suitable assays. Here, we have established a quantitative system based on the use of *Xenopus* egg extracts and human spermatozoa. This system provides novel possibilities for the functional characterization of human spermatozoa. Using clinical data we show that indeed this approach offers a set of complementary data for the functional evaluation of spermatozoa from patients.

## Introduction

Infertility is a global disease affecting 1 in 9 couples in developing countries. The introduction of ART such as *in vitro* fertilization (IVF)^1^ and especially intracytoplasmic sperm injection (ICSI)^2^, have helped to address infertility due to severe male factor; nevertheless, the live birth rate per cycle of IVF/ICSI is still less than 30%. One of the most important bottlenecks in IVF/ICSI cycles occurs during the first five days of development, i.e. up to blastocyst formation. Approximately 25% of the oocytes injected with a sperm arrest their development at day 1 due to fertilization errors^3,4^ while 25–30% of the remaining embryos are lost in culture during the first days of *in vitro* development^5,6^. Although some factors affecting embryo development, such as complex aneuploidies and severe sperm DNA fragmentation, have been identified, the systematic characterization of possible causes of embryo loss, at the molecular level, has been hampered by ethical and technical issues alike. The development of functional assays that recapitulate the process of human fertilization and early embryo development should improve our understanding of developmental arrest, allowing for better selection methods for gametes, and ultimately improving the success rate of IVF/ICSI.

After ovulation, the human oocyte is arrested at the metaphase of meiosis II (MII). When the sperm fuses with the oocyte, the oocyte is activated, meiosis resumes, and half of the sister chromatids are extruded into a second polar body^7^. The fertilised oocyte enters interphase before starting mitosis. In interphase, the male and female chromatin decondenses, replicates, and forms the male and the female pronuclei. Protamines are exchanged from the male chromatin for histones^8^. In addition, the basal body from the sperm recruits pericentriolar material (PCM) stored in the oocyte cytoplasm^9^. The centrosome in the fertilised oocyte generates an array of microtubules that mediates the male and female pronuclei movement and apposition, and prior to the first division of the zygote, the centrosome duplicates^7^.

The centrosome is the main microtubule organizing centre of the cell. It is formed by two centrioles, oriented perpendicularly to each other, surrounded by PCM^10^. In interphase, most microtubules are nucleated at the centrosome; it is therefore a main player in the intracellular organization as well as in cilia/flagella assembly, among other functions. During mitosis, two main pathways of microtubule assembly, the centrosomal and the chromatin-dependent pathways, cooperate to support spindle assembly^11^. The mitotic spindle is defined by the presence of the duplicated centrosomes that define the two poles and the orientation of the spindle thereby also defining the axis of cell division.

Spindle assembly and microtubule dynamics have been widely studied using the *Xenopus* egg extract system (XEE)^12^. *Xenopus* eggs are naturally arrested in MII by a CSF-dependent mechanism and do not contain centrosomes, resembling human oocytes. The cytoplasm of these eggs can be easily collected and centrifuged at low speed to obtain a large volume of cytoplasm arrested in MII-phase (CSF-XEE). The CSF-XEE can be manipulated in the test tube by addition of components (i.e. sperm) and the cell cycle state controlled to follow interphase and mitotic events associated with chromatin and microtubules. These events are physiologically relevant as they occur *in vivo* upon fertilization and culminate with the assembly of the first mitotic spindle^13^.

In the present study, we developed an *ex vivo* heterologous system based on the incubation of human spermatozoa in XEE in order to obtain functional information on the activity of the human sperm once placed in an oocyte cytoplasm environment. We further characterised the performance of semen samples from patients in this *ex vivo* heterologous assay. We show that this system provides functional data that may improve clinical diagnosis as well as our basic knowledge of the mechanisms that the spermatozoon triggers upon fertilization.

## Results

### Human spermatozoa assemble functional chromatin in *Xenopus* egg extracts

**Figure 1:**
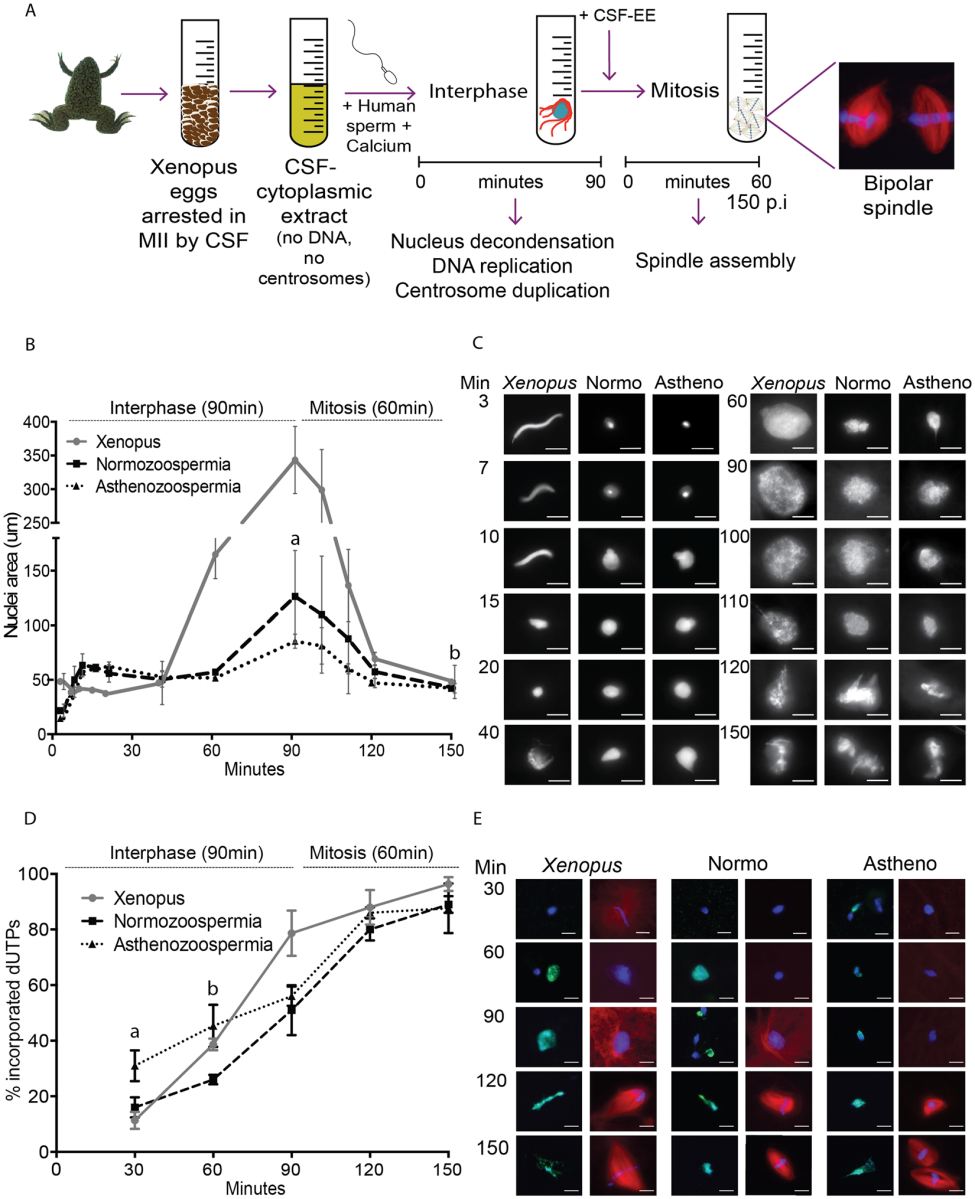
Human spermatozoa nuclei reorganise and replicate their DNA in XEE. (**A**) Schematic representation of the experimental design. 3,000 human (or *Xenopus*) sperm nuclei and calcium were incubated in the XEE to mimic fertilization. These incorporations induce the resumption of the cell cycle, first by 90 min of interphase followed by 60 min of mitosis. (**B**,**C**) Time course of sperm nucleus decondensation. *Xenopus* and human spermatozoa (normozoospermic and asthenozoospermic) incubated in XEE were retrieved at different time points along the 150 min of the experiment, to measure the nuclei area. The graph shows the area evolution in µm^2^ of the three different sperm samples. ^a^Significantly different p-value of 0.010. ^b^No significantly different p-value of 0.89. Dispersion data are given as SD. The images on the right are representative pictures of the nuclei decondensation per time point. Scale bar, 10 µm. (**D**,**E**) Time course of sperm DNA replication. *Xenopus* and human (normozoospermic and asthenozoospermic) spermatozoa, calcium and biotin-labelled dUTPs were incubated in the XEE and retrieved every 30-min. The graph shows the percentage of incorporated dUTPs per sample. ^a^Significantly different p-value of 0.017. ^b^Significantly different p-value of 0.013. Dispersion data are given as Standard Deviation (SD). The images are representative pictures of the dUTPs incorporation. dUTPs are in green, DNA in blue and microtubules in red. Scale bar, 10 µm.

We decided to test whether the XEE system could be suitable to perform functional studies on human spermatozoa and provide tools to address male infertility (Fig. 1A). One of the first and limiting events that occur after fertilization is the reorganization of chromatin. In interphase, the sperm chromatin decondenses and forms the male pronucleus; in mitosis, the chromatin condenses into chromosomes that align at the metaphase plate. We first addressed whether XEE could provide a system to study the chromatin-associated events that occur in the fertilised oocyte. We monitored chromatin decondensation and condensation of human normozoospermic samples incubated in XEE, using *Xenopus* sperm as control. The XEE extracts were induced to enter interphase by calcium addition. Samples were collected at different time points during the 90 min in interphase and then during the 60 min after cycling the extract back into mitosis. DNA decondensation during interphase and condensation during mitosis was quantified by measuring the area occupied by the chromatin. Human sperm chromatin decondensed gradually during interphase reaching a maximum area at the end of the 90 min interphase period, following a trend similar to the *Xenopus* sperm chromatin (Fig. 1B,C). As previously reported, the DNA of human sperm samples not pre-treated with DTT did not decondense when incubated in interphase XEE^14,15^. This suggests that the reduction of the disulfide bonds of protamines is necessary for their exchange by histones. The human sperm chromatin decondensation occurred in three phases: during the first 10 min the chromatin went through a rapid initial decondensation phase expanding to an area of 63.3 ± 10.5 μm^2^ (Fig. 1B,C). Subsequently, the chromatin condensed slightly over a period of approximately 30 min. After 40 min, the chromatin occupied an area of 50.3 ± 10.5 μm^2^. Towards the end of interphase (90-min), the chromatin had expanded into a round pronucleus with a maximum area of 126.4 ± 42.2 μm^2^. Although the overall pattern of decondensation of the human sperm chromatin was similar to that of *Xenopus*, the area occupied by the human chromatin was always smaller at the same time points. Indeed, it was 2.3 times smaller than the *Xenopus* sperm chromatin after 3 min in interphase XEE, and 2.7 times smaller at the end of interphase when the *Xenopus* sperm induced pronucleus occupied an area of 343.3 ± 49.8 μm^2^. Upon entry into mitosis by addition of CSF-XEE, both human and *Xenopus* chromatin condensed rapidly and aligned, forming the metaphase plate (Fig. 1B,C).

Having established the profile of chromatin decondensation/condensation for human normozoospermic samples during interphase and mitosis, we then looked at sperm samples with altered spermiogram. The chromatin decondensation profiles of asthenozoospermic samples was very similar to the normozoospermic ones during the first two phases, occupying areas of 62.4 ± 4.1 μm^2^ in phase 1 and 51.7 ± 2.7 μm^2^ in phase 2. More variability was observed in the last step of decondensation (area of 85.5 ± 6.5 μm^2^), which was significantly different compared with normozoospermic samples (p = 0.001). Chromatin from asthenozoospermic samples condensed upon entry into mitosis and formed the metaphase plate as efficiently as the chromatin from normozoospermic samples (p = 0.89) (Fig. 1B,C).

Overall we conclude that the XEE system is suitable to study human sperm chromatin decondensation in the interphase oocyte cytoplasm and its condensation into chromosomes during the first zygotic mitosis.

### The human spermatozoa DNA replicates in *Xenopus* egg extracts

After fertilization and during the first interphase the sperm chromatin replicates in preparation for the first mitosis of the zygote. We monitored the replication of human sperm DNA upon incubation in interphase XEE containing biotin-dUTPs. Immunofluorescence analysis at different time points of incubation showed that 16.0 ± 3.6% of the spermatozoa had incorporated biotin-dUTP in their chromatin after 30 min in interphase and 51.0 ± 9.0% after 90 min (Fig. 1D,E). In mitosis we detected that 89.0 ± 3.0% of the human chromatin groups had incorporated biotin-dUTPs suggesting that sperm chromatin replicated correctly. These percentages were similar to the control *Xenopus* spermatozoa. As expected, the global replication process appeared to be coordinated with the DNA decondensation phases. Asthenozoospermic and normozoospermic samples showed overall similar patterns of DNA replication, although there were significant differences during the first 60-min of the experiment (30 min p = 0.017; 60 min p = 0.013).

Altogether, these data suggest that the main sperm chromatin associated events (DNA decondensation and replication) that take place in the oocyte cytoplasm, can be visualised using XEE. This provides a novel system to evaluate patient samples. Here we assessed DNA decondensation and replication kinetics in human sperm samples showing abnormal motility and found that they did not present major chromatin decondensation or replication defects but there were significant differences in the kinetics of DNA replication.

### The human sperm basal body converts into a centrosome that nucleates microtubules in *Xenopus* egg extracts

**Figure 2:**
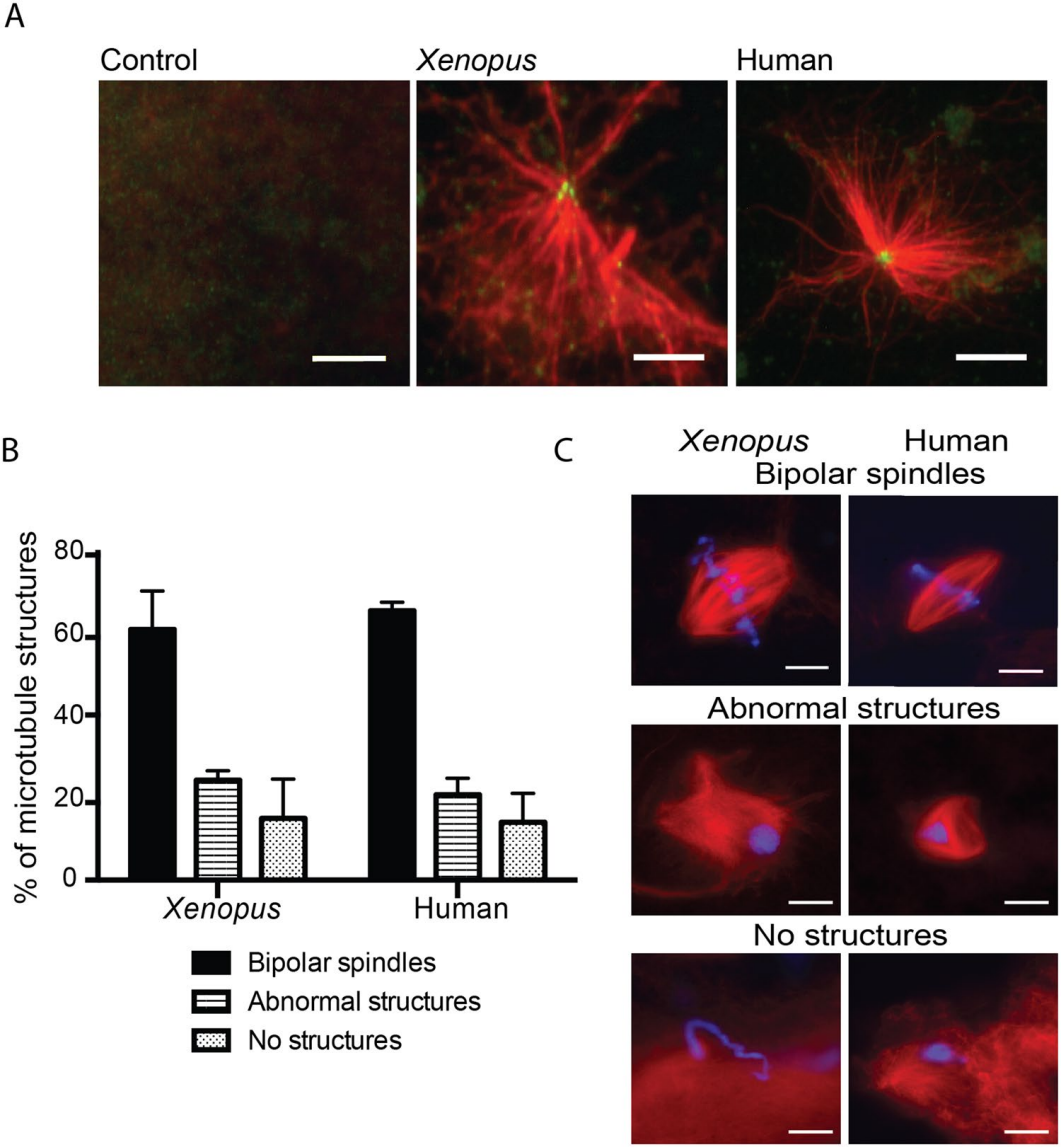
The human sperm basal body is converted into a fully functional centrosome in the oocyte cytoplasm. (**A**) Human sperm basal body actively nucleates microtubules in XEE. Immunofluorescence images of KI treated sperm samples incubated with XEE and pure tubulin. Microtubule asters are in red and centrioles in green. Scale bar, 5 µm. (**B**,**C**) Capacity of the *Xenopus* and human spermatozoa to assemble microtubule mitotic structures. The microtubules are in red and the DNA in blue. The structures associated with the DNA were classified as bipolar spindles, abnormal structures and no structures as shown in the images on the right. Scale bar, 10 µm. The graph on the left shows the analysis of 100 and 200 sperm nuclei for *Xenopus* and human samples respectively. No significant differences were found in any of the samples and microtubule structures. Dispersion data are given as Standard Deviation (SD).

Upon fertilization, the sperm basal body converts into the first centrosome of the new organism^16^. This basal body is an atypical centrosome with a ‘degenerated’ distal centriole and with little or no associated PCM that is recruited from the oocyte cytoplasm. Indeed, human spermatozoa incubated in pure tubulin *in vitro* did not nucleate microtubules. We then addressed directly the recruitment of PCM by the human sperm basal body centrioles using the XEE system. Human spermatozoa were stripped from PCM proteins and placed onto a coverslip. After incubation with XEE, the samples were washed and incubated with pure tubulin. Immunofluorescence analyses showed that the sperm centrioles did nucleate microtubules (Fig. 2A), suggesting that the human sperm centrioles had recruited PCM components from the XEE cytoplasm.

### Human spermatozoa trigger bipolar spindle assembly in *Xenopus* egg extracts

The correct formation of a bipolar spindle is not only an essential mechanism for the segregation of the genetic material in two daughter cells, but also for the development and survival of a healthy individual. We decided to test whether human spermatozoa can trigger spindle assembly in XEE. Human normozoospermic spermatozoa were pre-treated to loosen their membrane and incubated in XEE (Supplementary Table 1). The extract was sent into interphase by addition of Calcium and cycled back into mitosis by addition of CSF-XEE, as previously described for cycled spindle assembly assays with *Xenopus* sperm nuclei (Fig. 1A). After 60 min in mitosis, the samples were centrifuged onto coverslips, fixed and processed for fluorescence microscopy analysis. As a control, *Xenopus* sperm nuclei, also pre-treated to loosen their membrane, were processed in parallel. Microtubule structures associated with the sperm chromosomes were classified into three categories: (a) Bipolar Spindles (BP) having two focused spindle poles and the chromosomes well aligned at the metaphase plate; (b) Abnormal Structures (AB) consisting of disorganised microtubule arrays; and (c) No Structure (NS) when chromosomes had no associated microtubules. Human spermatozoa triggered bipolar spindle assembly in XEE as efficiently as the control *Xenopus* sperm nuclei; the proportion of bipolar spindles was 65.3 ± 3.2% for the human spermatozoa and 60.7 ± 10.5% for the *Xenopus* sperm nuclei (p = 0.53). The proportion of disorganised structures was also very similar for human spermatozoa (20.7 ± 4.0%) and *Xenopus* sperm nuclei (24.2 ± 1.7%; p = 0.26). Finally, 14.0 ± 7.0% (human) and 15.0 ± 9.5% (*Xenopus*) (p = 0.89) of the nuclei had no associated microtubules (Fig. 2B,C).

The morphology of the bipolar spindles formed around the human sperm chromosomes was also very similar to the control ones formed around *Xenopus* sperm nuclei. However, their length measured from pole to pole was 15.5% shorter (20 spindles per sample, p < 0.0001) (Supplementary Fig. 1).

We then tested whether sperm samples with different spermiograms can also form bipolar spindles in XEE, and whether they do it in different proportions. Asthenozoospermic (n = 4) and normozoospermic samples (n = 13) were tested for cycled spindle assembly in XEE as described above. We found that all samples triggered bipolar spindle assembly with no significant different efficiencies: 60.3 ± 7.6% of bipolar spindles for normozoospermic samples and 55.8 ± 4.6% for asthenozoospermic. Similarly no differences were found concerning the proportion of DNA masses with no associated microtubules (ln - no structures) (p = 0.82). However there was a significant difference in the proportion of abnormal structures in normozoospermic and asthenozoospermic samples (p = 0.010).

**Figure 3:**
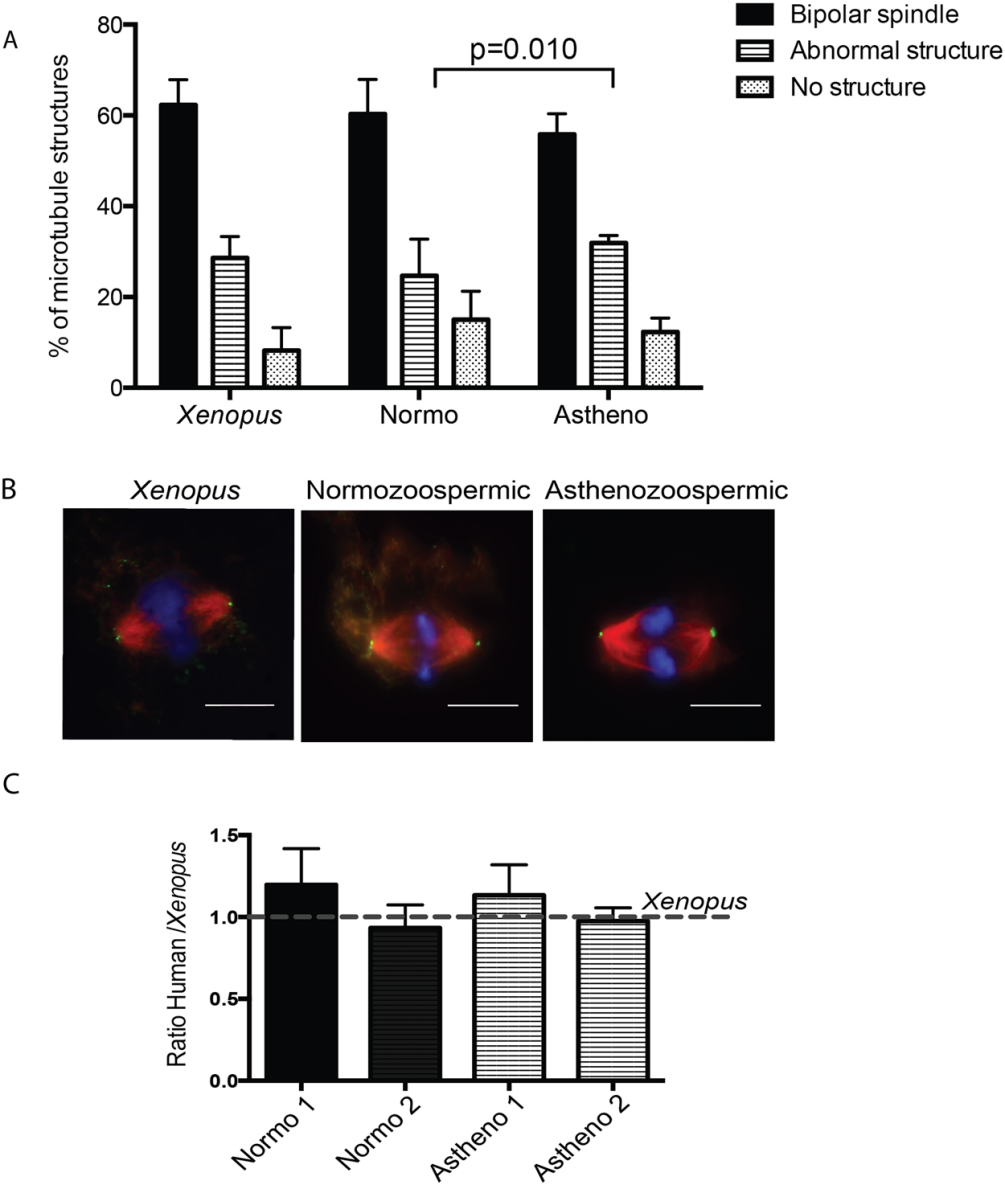
Human spermatozoa are able to assemble bipolar spindles in XEE independently of the sperm diagnosis. (**A**) Capacity to assemble microtubule mitotic structures of human spermatozoa samples with different diagnosis. The graph shows the percentage of bipolar spindles, abnormal structures and no structures per sample. Only significant differences were detected when comparing abnormal structures with normozoospermic and asthenozoospermic samples (p = 0.010). Dispersion data are given as Standard Deviation (SD). (**B**,**C**) Human basal body duplicates in XEE. Bipolar spindles assembled in XEE were treated with Nocodazole and processed for immunofluorescence. The number of bipolar spindles with centrosomes at both poles were analysed in 2 normozoospermic and 2 asthenozoospermic samples and normalised by the number of bipolar spindles with centrosomes at both poles in *Xenopus* spermatozoa. Dispersion data are given as Standard Deviation (SD). DNA is in blue, microtubules in red and the centrosome in green. Scale bar, 10 µm.

We then investigated whether centrioles were present at the poles of the spindles assembled by human spermatozoa by immunofluorescence microscopy^11^ (Fig. 3B,C). We could detect centrioles at the spindle poles with a similar frequency for both human and *Xenopus* samples. These data suggested that the human sperm centrioles had duplicated during interphase and correctly localised to the spindle poles. We obtained similar results for asthenozoospermic samples (Fig. 3B,C).

Altogether, our data show that human spermatozoa efficiently trigger the formation of bipolar spindles with centrosomes at their poles when incubated in XEE. They suggest that patient sperm samples with different diagnosis have different spindle assembly efficiencies not associated with centrosome defects.

### Predictive value of the spindle assembly test in *Xenopus* egg extracts in the context of clinical data

**Figure 4:**
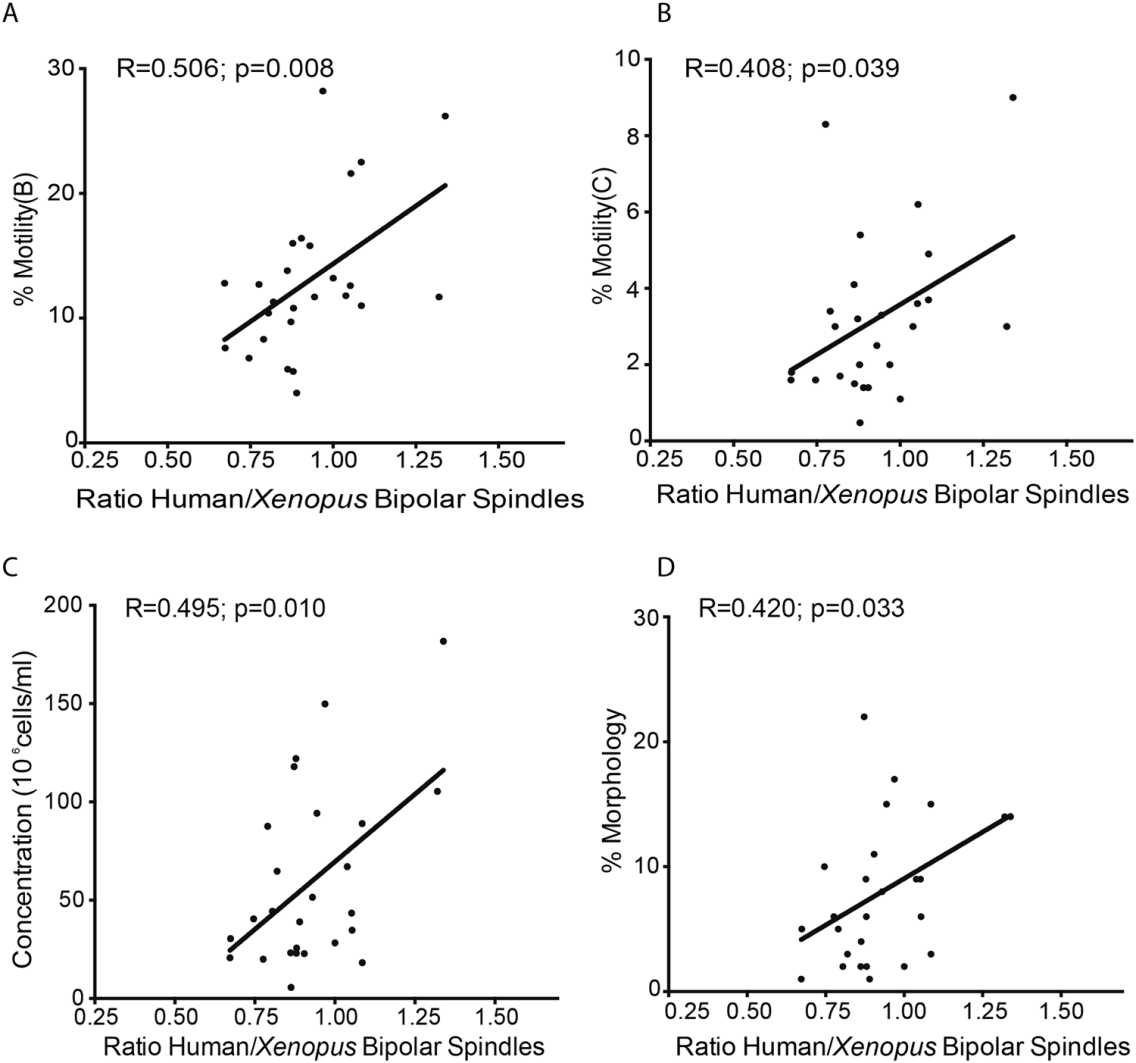
Human sperm samples have different capacities to trigger functional bipolar spindles. (**A**–**D**) The percentage of bipolar spindles per XEE was normalised by the percentage of bipolar spindles triggered by *Xenopus* spermatozoa in the same XEE. The average of bipolar spindles of each sample in the four XEE was correlated with each sperm sample characteristics and clinical outcomes for their respective IVF/ICSI cycle.

To determine whether the spindle assembly assay in XEE has any predictive value for IVF/ICSI for patients with different spermiogram results, cycled spindle assembly assays were performed for 26 individual samples (normozoospermic n = 13, asthenozoospermic n = 4, teratozoospermic n = 6, asthenoteratozoospermic n = 2, oligoasthenoteratozoospermic n = 1) (Supplementary Fig. 2). The assays were performed independently four times with different XEE. The ratio of bipolar spindles in each experiment was then quantified as described above and normalised to the results obtained from parallel experiments with control *Xenopus* sperm nuclei incubated in the same XEE. The averages from the four experiments showed a weak to moderate positive correlation between the ratio of bipolar spindle and three clinical parameters: sperm motility B (R = 0.506; p = 0.008) and C (R = 0.408; p = 0.039), concentration (R = 0.495; p = 0.010), and the percentage of morphologically normal spermatozoa (R = 0.420; p = 0.033) (Fig. 4A–D respectively).

Altogether, these results indicate that scoring spindle assembly efficiency of human sperm samples in XEE provides a novel test for assessing sperm functionality into the oocyte. Moreover, specific functional information can be obtained for difficult cases concerning chromatin physiological condensation/decondensation and replication as well as basal body conversion to a centrosome.

## Discussion

Although many oocytes fail to fertilise and a number of zygotes arrest their development during the first cell divisions after IVF/ICSI, the molecular causes of these failures remain difficult to investigate. Advances in this area are hampered by the lack of experimental systems that could provide functional information on the human gametes during and after fertilization. The present study describes a quantitative *ex vivo* heterologous system based on the use of XEE and human spermatozoa to evaluate the sperm capacity for driving microtubule nucleation and spindle assembly, providing a functional overview of the sperm behaviour during fertilization and early development. Although previous studies had used XEE to study human spermatozoa^17^, here we have expanded the array of functional analyses and taken them to a quantitative level to define different chromatin and microtubule-associated events that must occur for a successful fertilization and early development.

We have demonstrated that this heterologous system recapitulates the human sperm DNA decondensation-condensation and replication events as well as the assembly of functional spindle structures. Furthermore, we found that it is sensitive enough to detect small differences mainly in spindle assembly in different samples. Our data suggest that these differences could be used as functional markers for sperm samples. However, to obtain statistically significant differences due to the natural variability of the sperm samples and the XEE, each sperm sample has to be analysed at least 4 times, and this could be particularly problematic for the analysis of samples with severe oligozoospermia.

Some of the sperm factors reported to be related with poor embryo development are severe DNA damage^18^, epigenetic abnormalities^19^, severe aneuploidies^20^ and centrosome dysfunction^21^, among others. One of the earliest and critical events that occur in the zygote is the formation of a functional bipolar spindle to segregate correctly the genetic material into two daughter cells^22^. According to Simerly *et al*. about 25% of the fertilization failures observed in ART cycles are associated with defects in microtubule nucleation and organization^16^. Therefore, a system that could score the probability of assembly a functional spindle would inform about the chances of a successful IVF/ICSI cycle. We validated our functional system by scoring 26 patient samples with a variety of diagnosis for bipolar spindle assembly in XEE, and found that samples with a higher percentage of motile spermatozoa, higher concentration and a normal morphology trigger bipolar spindle assembly more efficiently. These results suggest that our quantitative system can be used to functionally diagnose sperm samples with IVF/ICSI cycles of already bad prognosis to propose the most convenient ART approach. They also provide some molecular evidences to the open question of whether the sperm selection criteria currently in use are effective clinically. Indeed, our results provide a validation of the current sperm selection criteria showing that normozoospermic samples with optimal parameters have higher probabilities to support fertilization and early development. We are currently expanding the analysis of samples including sperm with severe abnormalities, such as DNA damage, to further validate our system and to explore its clinically application.

To our knowledge this is the first assay that provides a correlation between semen diagnosis parameters and molecular data related to cell division in the oocyte. With this validation, we posit that our system will be useful to study several sperm dependent processes as well as the causes of male idiopathic infertility. For instance, it could be used to study spermatozoa that fail to activate the oocyte resulting in absent pronuclei, which represents the 15% and the 40% of the failed fertilizations after IVF/ICSI respectively; or the ones with abnormal pronuclei formation (approximately 20% of the failed fertilizations)^23^. In ART, the timing of pronuclear formation and cell division is an important indicator of fertilization and embryo quality. Our assay is amenable to check whether longer S-phases correlate with abnormal chromatin decondensation-condensation patterns and/or protamine exchange by histones, and whether these alterations affect later molecular events as bipolar spindle assembly. This would indicate whether the timing of pronuclei formation is a limiting factor for embryo development.

Several studies remarked the importance of the centrosome for pronuclei migration, syngamy and embryo cleavage^21,24,25^. Indeed, individual cases of severe asthenoteratozoospermia with abnormal head-to-tail attachment exhibit no microtubule nucleation from the centrosome once they are injected into a bovine oocyte^21^. Another study showed that the average rate of sperm aster formation was lower for spermatozoa from infertile patients injected in bovine oocytes than those produced by spermatozoa from fertile patients^26^. Our system could be very useful to analyse whether some idiopathic infertility cases are due to abnormal centrosome function as well as to obtain quantitative data for the basal body to centrosome transition and its microtubule nucleation activity *in vitro* for specific sperm samples.

Overall, we have developed and validated a quantitative heterologous system using human spermatozoa and XEE that mimics the events triggered by the human spermatozoa during fertilization. The system was validated using clinical data. It provides a quantitative approach to address molecular mechanisms that are very difficult to study in human oocytes due to ethical restrictions. Moreover, it reduces the bias originated by the heterogeneous human oocyte sample population, which in clinical studies cannot be ignored. It will be particularly interesting to use this system for cases of unexpected fertilization and early embryo development failures. The system could provide some molecular evidences to address these infertility cases.

## Materials and Methods

### Ethics

Ethical Committee permission to conduct this study was obtained from the Comité Étic d’Investigació Clínica CEIC-Parc de salut MAR (Barcelona). All procedures performed were in accordance with the ethical standards of the institutional research committees and with the 1964 Helsinki declaration, as revised in 2013. Written informed consent to participate was obtained from all participants prior to their inclusions in the study.

All the experiments involving animals were performed according to standard protocols approved by the ethical committee of the Parc de Recerca Biomèdica of Barcelona (PRBB), Barcelona, Spain.

All reagents were purchased from Sigma-Aldrich (Switzerland) or Fisher Scientific (United Kingdom) unless otherwise specified.

### Sperm quality and fertilization rate evaluation

Semen samples were evaluated after 30-min of liquefaction at room temperature with a computer-assisted semen analyser (Sperm Class Analyser, SCA Human Edition; Microptic S.L., Barcelona, Spain). The semen was classified according to the World Health Organization guidelines^27^. Normozoospermic semen samples contain all the semen analysed values above the reference limit (motility, cells with normal morphology and concentration), asthenozoospermic samples have the A + B motility below the reference limit, teratozoospermic samples contain a percentage of cells with normal morphology below the reference limit and oligozoospermic samples have a concentration below the reference limit. The ejaculate was then cryopreserved in straws with Sperm Cryoprotect II (Nidacon, Molndal, Sweden) and stored in liquid nitrogen up to sample preparation. Fertilization was assessed 14 to 19-h post-ICSI by visualization of two pronuclei and two polar bodies.

### Sperm preparation

Human sperm samples were thawed for 10-min at 37 °C. Then, the cryoprotectant was removed by washing the sample twice with HSPP buffer (250 mM sucrose, 15 mM Hepes pH 7.4, 0.5 mM spermidine, 0.2 mM spermine and protease inhibitors) and centrifuged at 850 g for 10 min at room temperature. After some initial optimization, (Supplementary Table 1) sperm samples pellets were treated with HSPP buffer containing 1 mM of DTT during 10 min at room temperature. Next, the same volume of HSPP buffer containing 1 mM of DTT and 0.5% Triton X-100 was added to the solution and kept in agitation for 30 min at room temperature. The reaction was stopped adding HSPP – BSA 3% in excess and chilled on ice for 10-min followed by centrifugation at 850 g for 10 min at 4 °C. The pellet was washed with HSPP – BSA 0.3% and then diluted with HSPP – 0.3% BSA – 30% glycerol and adjusted to 2 × 10^7^ cells/ml before freezing. *Xenopus* spermatozoa were treated as previously described^28^.

### Egg extract and spindle assembly

*Xenopus laevis* frogs were purchased from Nasco and used at an age between 1 and 3 years. Preparations of fresh CF-arrested *Xenopus* egg extract (CSF-XEE) and cycled spindle assembly reaction were performed as previously described^29^. Briefly, *Xenopus laevis* frogs were stimulated with 100 and 1,000 I.U. PMSG and HCG respectively to lay eggs arrested at MII. The eggs were centrifuged at 10,000 g at 4 °C and then the cytoplasmic layer was retrieved using a 1 ml syringe and an 18G needle taking care not to disturb the black pellet and the lipid layer. To analyse spindle assembly in cycled XEE, 3,000 sperm nuclei (human or *Xenopus*) together with 0.4 mM of Ca^2+^ and ≈0.2 mg/ml of Rhodamine-labelled tubulin (to visualise microtubules) were added to the CSF-XEE and placed at 20 °C to release the XEE into interphase (90 min). Then, the interphase XEE was cycled back to mitosis by adding the same volume of CSF-XEE (60 min; total time of 150 min) (Fig. 1A). After 60 min, spindle assembly was monitored by “squashing” 1 µl of the reaction and 3 µl of fix solution (11% formaldehyde, 48% glycerol, 1 µg/ml Hoechst 33342 (1 µg/ml, Invitrogen) in CSF-XB buffer (10 mM Hepes, 100 mM KCl, 0.1 mM CaCl_2_, 2 mM MgCl_2_, 50 mM sucrose, 5 mM EGTA)) between a 18 × 18 mm coverslip an a glass slide. For immunofluorescence analysis, the spindle reaction was diluted in spindle dilution buffer (30% glycerol, 1% Triton-X100, 1x BRB80 (80 mM K-pipes, pH 6.8, 1 mM MgCl_2_, 1 mM Na_3-_EGTA)) and centrifuged (4,000 g for 20 min at room temperature) on a coverslip through a 4 ml spindle cushion buffer (40% glycerol, 1x BRB80). The coverslips were recovered, washed with PBS, fixed for 10-min in cold methanol and the DNA was stained with Hoechst 33342 (1 µg/ml, Invitrogen) for 30 min. Samples were visualised with an inverted DMI-6000 Leica wide-field fluorescent microscope using a 63x objective. The same microscope and image processing was used for all the experiments.

### Decondensation and replication assays

To analyse the decondensation dynamics of the spermatozoon nucleus, 2 µl of cycling XEE were “squashed” with 3 µl of fixing solution between a 18 × 18 mm coverslip and a glass slide. The area of individual sperm nucleus was analysed for each time point using Fiji (ImageJ software, NIH, USA). For the replication assay, 40 µM of biotin-dUTPs (R0081, ThermoFisher) were added to the cycling XEE. 10 µl of the mix was taken every 30 min and fixed for 1 hour at room temperature in 200 µl of XB (10 mM Hepes, 100 mM KCl, 0.1 mM CaCl_2_, 1 mM MgCl_2_, 50 mM sucrose) containing 4% formaldehyde. The samples were centrifuged at 2,500 g through a 0.7 M sucrose cushion in XB during 20 min onto a glass coverslips and post-fixed for 4 min in cold methanol at −20 °C. Finally, Streptavidin-Alexa Fluor 488 conjugate antibody (S11223, ThermoFisher) and Hoechst 33342 (1 µg/ml, Invitrogen) diluted with PBS 1% BSA were loaded on the coverslip for 1 hour at room temperature. The sample was then washed twice with PBS 0.1% Tween 20 and mounted with Mowiol before observation under the fluorescence microscope. 20 spermatozoa per condition and time point were analysed in three independent experiments.

### Tubulin and centrosome detection

Rhodamine-labelled tubulin was added to the XEE to visualise the nucleation of microtubules. Centrioles of the bipolar spindles were detected by adding Nocodazole (0.5 uM, 20 min at 20 °C, M1404 Sigma) to the XEE before spinning them down^11^. Briefly, right after the pelleting of the samples, they were blocked with 5% BSA 0.1% Triton X-100 in PBS for 50 min at room temperature, and the monoclonal mouse anti-centrin antibody (20H5, Millipore, 1:2,000) was then used to immunodetect the centrosomes. The percentage of detected centrioles at both poles, one pole and no centrioles was quantified for *Xenopus*, human normozoospermic and asthenozoospermic sperm samples in four independent experiments.

### Centrosome complementation assay

To assess the capacity of the human centrioles to recruit PCM and to nucleate microtubules, we used a previously published protocol with minor modifications^30^. Briefly, 100,000 human spermatozoa were incubated with 4 M KI in 1xBRB80 and placed onto a poly-lysine coated glass coverslip during 10 min at 30 °C in a humidified chamber. The adhered sample was washed by pipetting three times with 60 µl of Hepes block buffer (100 mM KCl, 10 mg/ml BSA, 1 mM β-mercaptoethanol) before incubation with 60 µl of XEE for 10 min at 30 °C and then washed briefly three times with 60 µl of TDB wash buffer (1x BRB80, 10% Glycerol, 1 mM GTP and 10 mg/ml BSA). Twenty-five µl of unlabelled brain cow tubulin at 2 mg/ml in TDB (1x BRB80, 10% Glycerol, 1 mM GTP) was added to the sample for 10 min. Next, the samples were fixed for 3-min in 1% glutaraldehyde and post-fixed for 3 min in cold methanol at −20 °C. The coverslips were quenched with 0.1% sodium borohydride in TBS (Tris buffer saline) for 7 min. Microtubule and centrosomes were visualised by immunostaining with a rabbit monoclonal anti-beta-tubulin antibody (ab6046 Abcam, 1:200) and a mouse polyclonal anti-centrin antibody (20H5 Millipore, 1:2,000).

### Statistical analyses

To apply the statistical analyses, first of all we evaluated the normality of the samples. Visual inspection of the distribution using Q-Q plot (quantile-quantile plot) and Shapiro-Wilk test were performed. Continuous variables normally distributed were analysed using Student t-test or one-way ANOVA followed by Bonferroni’s post-hoc comparisons test. For non-normal distributed variables, logarithm transformation was done before subsequent analyses (i.e. no structures), and if the log transformed variable (i.e. DNA decondensation) was still not normal, non-parametric tests (Mann-Whitney test) were applied. Dispersion data in all the analyses is given as Standard Deviation (SD). These analyses were performed using the statistical packages Prism 6 (GraphPad Software, La Jolla, CA) and IBM SPSS version 22.0 (Statistical Package for the Social Sciences. New York, USA).

For each sperm sample triggering bipolar spindles in XEE, 100 and 200 sperm nuclei were counted for *Xenopus* and human samples, respectively in individual XEE (a total of 4 independent XEE incubations with a total number of 400 and 800 *Xenopus* and human sperm nuclei analysed, respectively). The microtubule structures associated with the spermatozoa DNA were classified as: bipolar spindles, abnormal structures or no structures.

For spindle length measurements, the length of 20 spindles per sample was analysed using a straight line from ImageJ software.

To analyse spindle assembly by sperm patients samples, the percentage of bipolar spindles was normalised on the percentage of *Xenopus* sperm bipolar spindles in the same XEE. Semen parameters (motility (A, B, A + B, C and D), morphology, concentration, number of cells and volume) and fertilization rate were described for each sperm patient sample and associated IVF/ICSI cycle. The linear relationship between bipolar spindles and the semen parameters was assessed using Pearson correlation, as well as the relationship between bipolar spindles and fertilization rate. A Pearson’s R coefficient values of 0.3 to 0.5 were considered as weak correlation and values of 0.5 to 0.7 as a moderate correlation.

For all the analyses the statistical threshold was set at p < 0.05.

## Electronic supplementary material

Supplementary Information

## Data Availability

Data from this work are available upon request.
