# Supplementary material for: Functional Analysis of Human Pathological Semen Samples in an Oocyte Cytoplasmic Ex Vivo System
Source: Sci Rep. 2018 Oct 18;8:15348. doi: 10.1038/s41598-018-33468-x (PMC6194145; doi:10.1038/s41598-018-33468-x)
Supplement: Supplementary file 1 — Supplementary Information [file 41598_2018_33468_MOESM1_ESM.docx]

**FUNCTIONAL ANALYSIS OF HUMAN PATHOLOGICAL SEMEN SAMPLES IN AN OOCYTE CYTOPLASMIC *EX VIVO* SYSTEM**

Farners Amargant^1,2^, Désirée García^3^, Montserrat Barragán^2^, Rita Vassena^2*^, Isabelle Vernos.^1,4,5*^

**Affiliations:**

^1^ Cell and Developmental Biology Programme, Centre for Genomic Regulation (CRG), Barcelona Institute of Science and Technology, Doctor Aiguader 88, 08003 Barcelona, Spain.

^2^ Clínica EUGIN, Travessera de les Corts 322, Barcelona, 08029, Spain.

^3^ Fundació EUGIN, Travessera de les Corts 314, Barcelona, 08029, Spain.

^4^ Institució Catalana de Recerca I Estudis Avançats (ICREA), Passeig de Lluis Companys 23, 08010 Barcelona, Spain

^5^ Universitat Pompeu Fabra (UPF), Barcelona, Spain

*To whom correspondence should be addressed:

rvassena@eugin.es, [isabelle.vernos@crg.es](mailto:isabelle.vernos@crg.es)

**
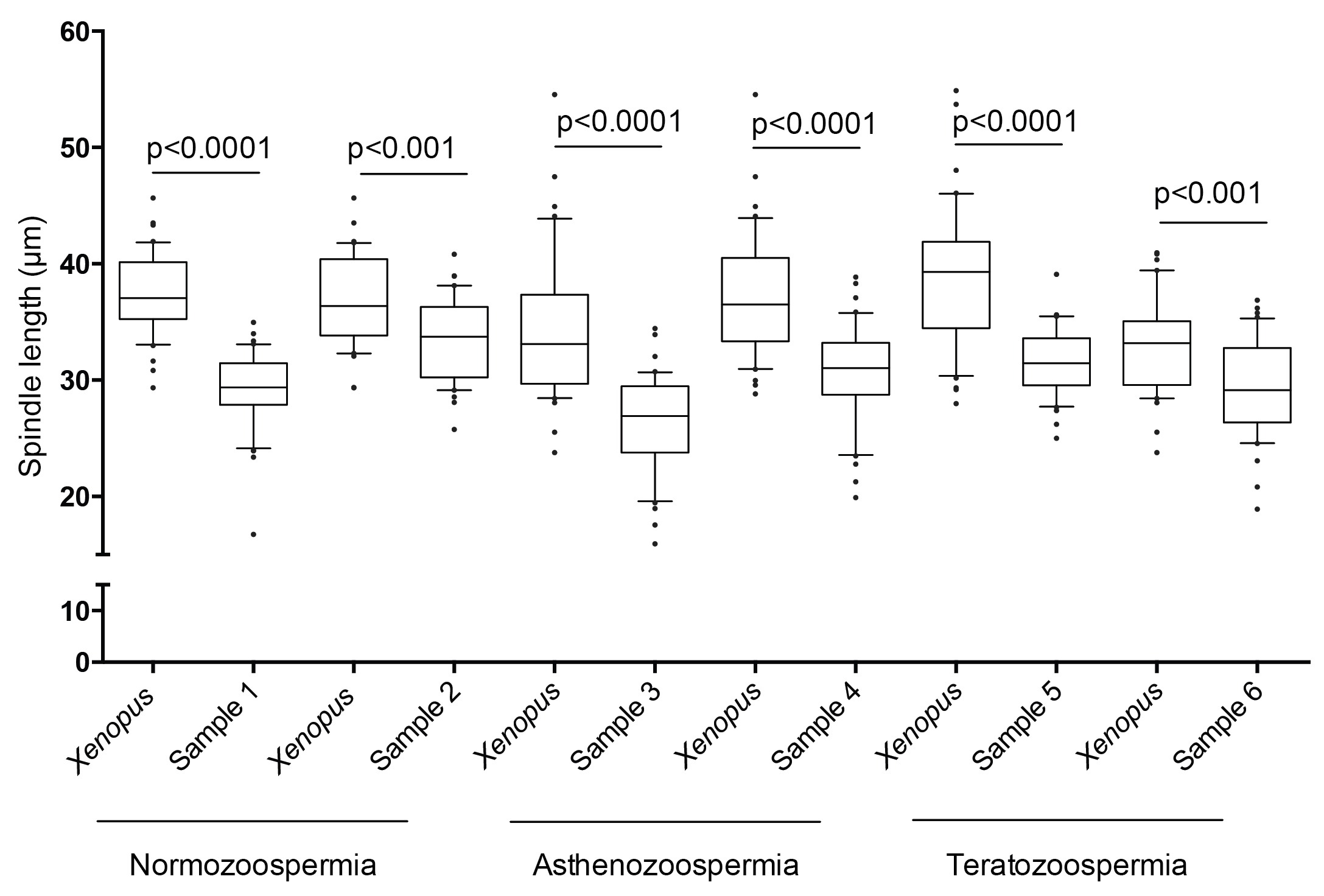
**

**Supplementary Figure 1**

**Bipolar spindles assembled around human sperm are smaller than those assembled around *Xenopus* sperm.** The graph shows the length of bipolar spindles assembled by Xenopus sperm and normozoospermic, asthenozoospermic and teratozoospermic human samples.

**
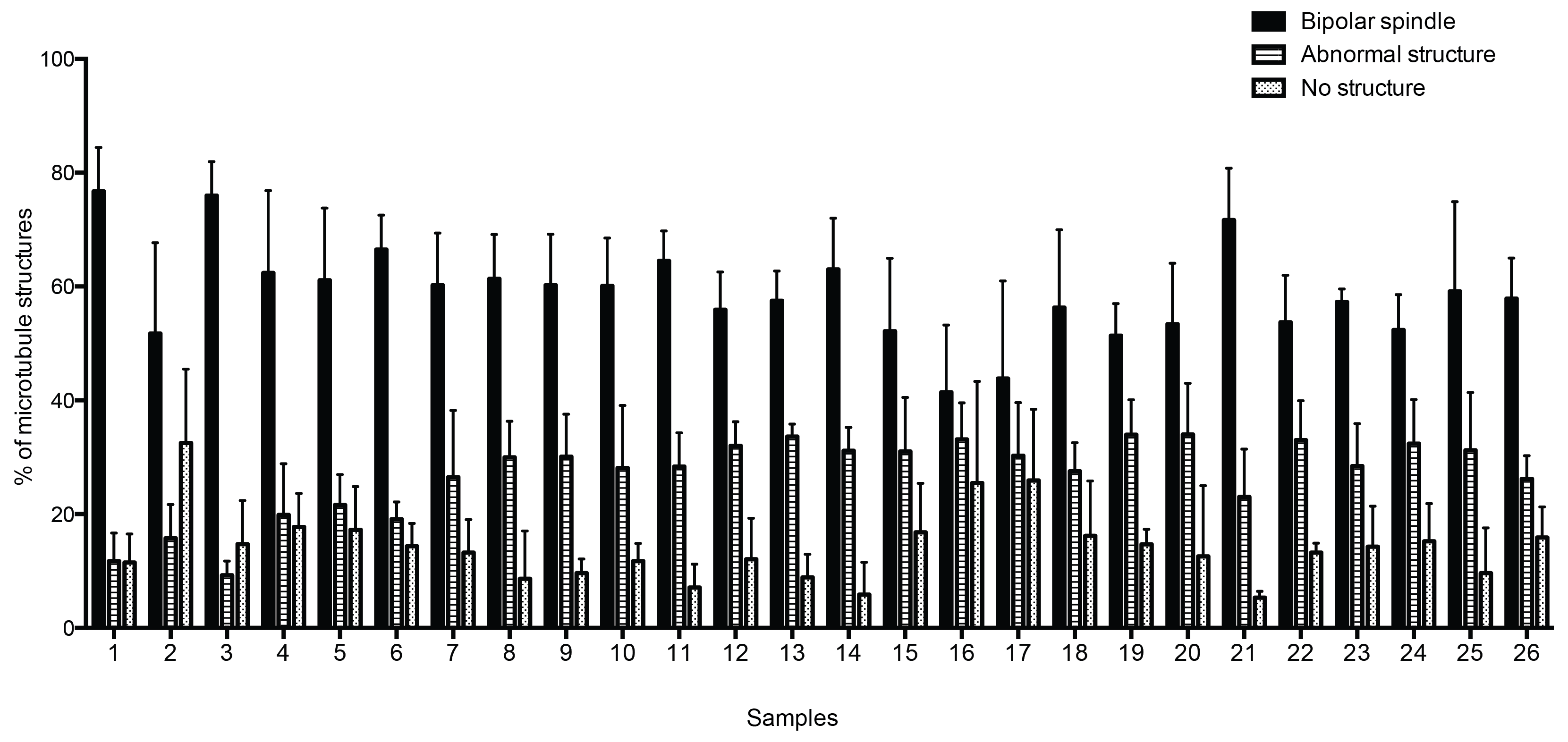
**

**Supplementary Figure 2**

**Microtubule structures formed upon incubation of individual human semen samples with different diagnosis in XEE.** The graph shows the percentage of bipolar spindles, abnormal structures and no structures per semen sample.

**Supplementary Table 1**

**Conditions tested to optimise the human sperm membrane destabilization**

| CONDITION | BIPOLAR SPINDLES (%) | ABNORMAL STRUCTURES (%) | NO STRUCTURES (%) |
| --- | --- | --- | --- |
| Xenopus | 60.8 | 24.2 | 15.0 |
| Human not treated | 2.3 | 0.9 | 96.8 |
| Lysolecithin 1.5% | 9.0 | 2.2 | 88.8 |
| Lysolecithin 1.5% + 1mM DTT | 57.3 | 22.0 | 20.7 |
| Triton 0.05% | 13.3 | 1.6 | 85.1 |
| Triton 0.25% | 2.2 | 5.0 | 92.7 |
| Triton 0.25% + 1mM DTT | 65.3 | 20.7 | 14.0 |
| NP40 0.05% | 5.4 | 3.6 | 91.0 |
| NP40 0.25% | 12.4 | 3.7 | 83.9 |
| NP40 0.25% + 1mM DTT | 65.7 | 18.7 | 15.7 |
| 1mM DTT | 50.7 | 20.0 | 29.3 |
